# In-Plane MEMS Shallow Arch Beam for Mechanical Memory

**DOI:** 10.3390/mi7100191

**Published:** 2016-10-18

**Authors:** Md Abdullah Al Hafiz, Lakshmoji Kosuru, Abdallah Ramini, Karumbaiah N. Chappanda, Mohammad I. Younis

**Affiliations:** 1 Computer, Electrical and Mathematical Science and Engineering Division, King Abdullah University of Science and Technology (KAUST), Thuwal 23955-6900, Saudi Arabia; abdullah.hafiz@kaust.edu.sa; 2 Physical Science and Engineering Division, King Abdullah University of Science and Technology (KAUST), Thuwal 23955-6900, Saudi Arabia; lakshmoji.kosuru@kaust.edu.sa (L.K.); abdallah.ramini@kaust.edu.sa (A.R.); karumbaiah.nanaiah@kaust.edu.sa (K.N.C.)

**Keywords:** in-plane MEMS, shallow arch, bistability, mechanical memory

## Abstract

We demonstrate a memory device based on the nonlinear dynamics of an in-plane microelectromechanical systems (MEMS) clamped–clamped beam resonator, which is deliberately fabricated as a shallow arch. The arch beam is made of silicon, and is electrostatically actuated. The concept relies on the inherent quadratic nonlinearity originating from the arch curvature, which results in a softening behavior that creates hysteresis and co-existing states of motion. Since it is independent of the electrostatic force, this nonlinearity gives more flexibility in the operating conditions and allows for lower actuation voltages. Experimental results are generated through electrical characterization setup. Results are shown demonstrating the switching between the two vibrational states with the change of the direct current (DC) bias voltage, thereby proving the memory concept.

## 1. Introduction

The quest for mechanical computation is a century old, and can be traced back to at least 1822, when Babbage presented his concept of a difference engine [1]. Although subsequent developments in the fields of electronic transistors [2] and magnetic storage [3,4] outperformed the mechanical approach in computation, this field has reemerged (at least in the research community [5,6,7,8,9,10,11]) due to recent advancements in micro/nano-fabrication and measurement techniques. Various physical phenomena can be utilized to store data in memory devices. Instead of storing the data in the form of a packet of electric charge, the single bits of data are retained by a mechanically bistable beam having two stable states of vibration; this initiated interest in the development of microelectromechanical systems (MEMS) memory devices [12]. A memory device in the form of a micro-mechanical device is an alternative memory element for storing digital electronic signals. Micro-mechanical devices can be fabricated on a semiconductor substrate, such as silicon. The same manufacturing steps used to fabricate electronic elements in an integrated circuit can be used.

One of the key components in computation is a memory element, which is supposed to work as a two-state system representing two different states of the memory device, irrespective of the mode of operation (static or dynamic). In the case of a static mechanical memory device, the logical information is preserved in one of the two stable spatial states [12,13,14,15]. The mechanical element only moves during the change in the state of the memory. Consequently, the write operation is performed by applying an external force. Once the desired state is reached, it does not require continuous forcing to maintain its state. Several designs based on carbon nanotubes and nanowires have been proposed for static memory devices [13,14]. Static devices have a large on/off ratio and simple operation mechanism. However, they have limitations, such as lesser write/read speed and degradation of the contact area due to large displacement. Another approach to achieve bistability in MEMS is the utilization of its dynamic motion in nonlinear behavior. The advantages of dynamic MEMS over static devices are small vibration amplitudes and low switching time. In contrast, a dynamic mechanical memory utilizes two different vibrational states accessible within the hysteretic region of a nonlinearly resonating micro/nano resonator to constitute the memory states [5,6,7,8,9]. These micro/nano-electromechanical systems resonators use resonant motion, in which the compliant structures undergo relatively small displacements during the memory operation. Thus, they enable high-speed operation with high durability. Stiction and friction of the beams plays an important role in the failure of static MEMS devices. An advantage of using a dynamic MEMS approach is the avoidance of these shortcomings.

A MEMS resonator substantially exhibits nonlinear responses at large excitation forces. In this case, frequency response curves bend toward higher or lower frequencies due to a hardening or softening effect [16,17,18]. This means that a nonlinear MEMS resonator can have two stable periodic states and an unstable periodic state at fixed operating conditions in the hysteresis region [19]. Several applications, such as very-high frequency resonators, mass detection, and up-converter amplifiers based on the nonlinear behavior of nanoelectromechanical systems/microelectromechanical systems (NEMS/MEMS) resonators have been published [20,21,22,23]. In addition to the experimental investigations, the literature regarding the theoretical studies is quite extensive [24,25,26,27,28,29,30,31,32,33,34,35,36,37,38]. Abdel-Rahman and Nayfeh [24] studied the dynamic response of a MEMS resonator under superharmonic and subharmonic electric actuations by considering the geometric and electric force nonlinearites, and employed the method of multiple scales to solve the equation of motion. Nayfeh and Younis [25] examined the nonlinear dynamic behavior of electrostatically actuated microbeams using a shooting method along with the Galerkin technique.

Many theoretical studies contributed to the analysis of the nonlinear static and dynamic behavior of geometrically imperfect beams and microbeams [30,31,32,33,34,35,36,37,38,39,40,41,42]. Due to the inability of classical theories to model size-dependent behavior of microscale structures, several size-dependent theories, such as the couple stress theory [30], the strain gradient theory [31], and the modified couple stress theory [32,33,34,35] have been developed. Wang et al. [31] developed a microscale Timoshenko beam model based on the strain gradient elasticity theory. Farokhi and Ghayesh [32] investigated the thermo-mechanical three-dimensional nonlinear size-dependent behavior of a Timoshenko microbeam for both initially straight and slightly curved microbeams. Recently, the size-dependent Euler–Bernoulli and Timoshenko beam models have been further extended to their nonlinear counterparts by taking the von Karman’s nonlinear strains into account. Ghayesh and Farokhi [33,36] used the Kirchoff plate theory to investigate the nonlinear dynamics of a rectangular microplate, and obtained the frequency–response and force-–response curves. Saghir and Younis [37] investigated the static and nonlinear dynamic behavior of electrostatically-actuated microplates using a reduced order model based on the von Karman equations. Lou et al. [38] investigated the nonlinear bending and free vibration behavior of simply supported functionally graded microplates based on a general size-dependent four variable refined plate model. Semnani et al. [39] studied the free flexural vibration characteristics of functionally graded microbeams with geometric imperfection, taking into account the size effect phenomenon based on modified couple stress theory. Shafiei et al. [40] investigated the size-dependent nonlinear vibration behavior of a Euler–Bernoulli functionally graded microbeam made of porous material. The bistability of MEMS resonators in the hysteretic regime can be effectively used for the implementation of mechanical memory application. Badzey et al. [5] reported for the first time a mechanical “1” bit memory based on a nonlinear NEMS resonator. Guerra et al. [6] presented a silicon nano-resonator controlled by a novel phase modulation scheme to build a controllable resonant switch. Noh et al. [7] proposed and demonstrated a direct current (DC) modulated memory device based on optical detection. Vestra et al. [8] investigated the nonlinear dynamics of a micro-cantilever driven by a piezo actuator, and demonstrated memory operation utilizing an optical detection technique. They utilized the hardening behavior of the fundamental mode of a micro-cantilever due to its geometric nonlinearity for the implementation of the memory device. Uranga et al. [9] demonstrated a NEMS micro-cantilever and clamped–clamped microbeam based mechanical memory devices fabricated on commercial complementary metal-oxide-semiconductor (CMOS) technology. Recently, Yao et al. [11] developed a logic-memory device and a binary counter in single and coupled MEMS resonators, respectively, based on feedback control of the nonlinear motion of the micro-resonators.

Apart from the work of Badzey et al. [5], most of the above-mentioned works utilized the hardening nonlinearity of NEMS/MEMS micro-resonators (clamped–clamped straight microbeam and cantilever beams) for the demonstration of mechanical memory operations. Here, we exploit the softening behavior in an arch-shaped beam for memory application. Unlike electrostatically actuated cantilever microbeams, where the softening nonlinear behavior is solely caused by the DC bias voltage, an arch shaped clamped–clamped microbeam shows softening nonlinearity due to its initial curvature [41,42,43,44,45,46]. Hence, the nonlinearity in this case is mainly of mechanical origin, and thus is almost independent of the DC bias (does not put any restriction on the amount of bias needed to trigger the nonlinearity), and can even be actuated using other methods, such as piezoelectric. Unlike a micro-cantilever, a clamped–clamped arch beam exhibits softening nonlinearity at very small motion, is mechanically robust, and if necessary can be actuated at higher voltages, without pull-in. Further, exciting micro-cantilever beams into a hardening behavior (as demonstrated in the literature) requires driving them into significantly large motion compared to their thickness, which adds constraints on the fabrication and boosts their power demands.

The utilization of electrostatic transduction for memory operation promises the prospect of fully integrated on-chip system development. In a previous work [47], we demonstrated that an arch beam can effectively be used to construct all the basic logic gates. Hence, the next step to establishing a complete integrated computational hardware is to use the same structure for memory applications.

In this work, we present a silicon in-plane microbeam, intentionally fabricated as a shallow arch shape, operating as a two-state system at room temperature and under modest vacuum conditions with electrostatic actuation and detection. The softening nonlinearity of quadratic type owing to the arch initial curvature is utilized to constitute a hysteresis frequency band that can be modulated by either the drive amplitude or the DC bias voltage. We employ instantaneous change in the DC bias voltage amount as our modulation signal, and show a memory operation by switching the resonator between the two stable periodic vibrational states within the bifurcation branch.

## 2. Device and Experimental Setup

The arch beam is fabricated on a highly conductive Si device layer of silicon on insulator (SOI) wafer by a two mask process using standard photo-lithography, E-beam evaporation for metal layer deposition for the actuating pad, deep reactive ion etch (DRIE) for silicon device layer etching, and vapor HF etch to remove the oxide layer underneath of the resonating structure. It consists of a clamped–clamped arch shaped beam with two adjacent electrodes to electrostatically induce the vibration and detect the generated alternating current (AC) output current due to the in-plane motion. The dimensions of the curved beam are 500 µm in length, 3 µm in width, and 30 µm in thickness. The gap between the actuating electrode and the beam is 8 µm at the clamped ends and 11 µm at the mid-point, due to its 3 µm initial curvature. We note that, unlike surface micro-machined clamped–clamped arch beams where the buckling of the structural layer upon release due to compressive residual stress may often produce uncontrollable curved beam configuration, the fabrication process utilized in this work for the in-plane arch is controllable and reproducible, with minimal constraints from residual stresses.

**Figure 1:**
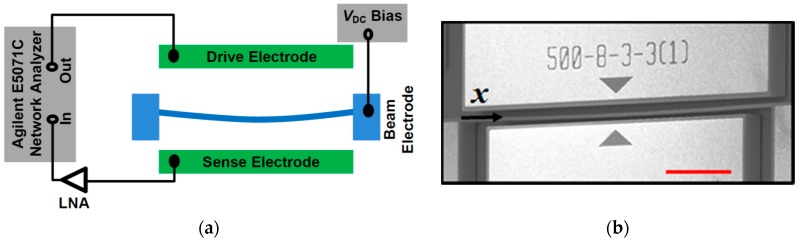
(**a**) Block diagram of the two-port electrical transmission measurement; (**b**) an scanning electron microscope (SEM) image of the arch beam resonator. The scale bar is 100 µm. LNA: low noise amplifier.

Figure 1a shows the block diagram of the two-port electrical transmission measurement configuration for electrostatic actuation and sensing. The drive electrode is provided with an AC signal from the network analyser (Agilent E5071C, Keysight Technologies, Santa Rosa, CA, USA) and the beam electrode is biased with a DC voltage source, which is also used to modulate the nonlinear resonance. The output current induced at the sense electrode is coupled with a low noise amplifier (LNA) whose output is connected to the network analyser input port. Figure 1b shows an scanning electron microscope (SEM) image of the fabricated clamped–clamped arch beam.

## 3. Softening Nonlinearity in Arch Microbeam

**Figure 2:**
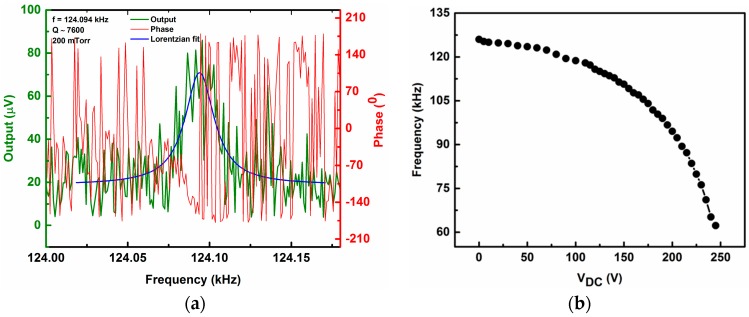
(**a**) The linear resonance frequency and phase response of the arch beam at *V*_DC_ = 10 V and *V*_AC_ = −30 dBm. The magnitude curve is fitted with Lorentzian fit; (**b**) Theoretically predicted frequency response of the arch beam with applied direct current (DC) bias voltage.

At small driving voltages, the responses of the arch beam fit the Lorentzian linear shape. Figure 2a shows a frequency–response curve measured at *V*_DC_ = 10 V and *V*_AC_ = −30 dBm (7.07 mV_rms_), indicating a linear resonance frequency near 124.094 kHz with a quality factor *Q* = 7600 at a pressure of 200 mTorr. The variation of resonance frequency with applied DC bias voltage for the same arch beam is predicted theoretically (shown in Figure 2b). As the drive amplitude is increased, the beam response enters the nonlinear regime. Next, we prove the inherent quadratic nonlinearity of the arch regardless of its actuation method (i.e., whether there is a DC electrostatic force or not), which shows a softening nonlinear response. The nonlinear equation governing the transverse motion *w*(*x*,*t*) of the arch beam based on no axial inertia can be written as [41,42,43,44,45]:

(1) $$EI\frac{\partial^{4}w}{\partial x^{4}}+\rho A\frac{\partial^{2}w}{\partial t^{2}}+c\frac{\partial w}{\partial t}=\left(\frac{\partial^{2}w}{\partial x^{2}}+\frac{d^{2}w}{dx_{0}^{2}}\right)\left(\frac{EA}{2L}\int_{0}^{L}\left\{{\left(\frac{\partial w}{\partial x}\right)}^{2}+2\left(\frac{\partial w}{\partial x}\frac{\partial w_{0}}{\partial x}\right)\right\}dx\right)-F$$

where *x* is the spatial position, *t* is time, and *w*_0_ is the initial curvature of the arch. The arch has a Young’s modulus *E*, a material density ρ, and is assumed to have a rectangular cross-sectional area *A* and a moment of inertia *I*. Additionally, it is subjected to a viscous damping coefficient *c*. The arch is of length *L*, width *b*, and thickness *h*. It is subjected to an electrostatic force *F*. It is clear from the integral term of the Equation (1) that the stiffness depends nonlinearly on the displacement, which is a major source of nonlinear behavior. To distinguish the nonlinearities of the arch from that of the electrostatic force, we will assume the force is constant (full modelling of its effect can be found in [41,42,43,44,45,46]). The initial shape of the arch is assumed to be

(2) w0=−b02[1−cos(2πxL)]

where *b*_0_ is the initial curvature at the middle of arch. Using a first-mode approximation with the arch mode shape [45,48] in the Galerkin procedure [16], dropping damping, and after normalization, the non-dimensional equation governing the modal amplitude *u* of the first mode can be written as

(3) $$\overset{\cdot \cdot }{u}\left(t\right)+\omega_{0}^{2}u\left(t\right)+\alpha_{2}u^{2}(t)+\alpha_{3}u^{3}(t)=f$$

where *f* is the projected modal force, $\omega_{0}^{2}\;=\;1557.38$ is the non-dimensional frequency squared, and α_2_ = −3749.87 and α_3_ = 5283.173 are the quadratic and cubic nonlinearity coefficients, respectively.

Following [49], it turns out that this system with quadratic and cubic nonlinearities can exhibit softening or hardening behaviour, depending on whether or not the quadratic nonlinearities are dominant over the cubic nonlinearities (full analysis of this can be found in [42]. According to [49], if the sign of the effective nonlinearity coefficient α is positive, the behavior is hardening, otherwise the behavior is softening. According to [49],

(4) $$\alpha =\alpha_{3}-\frac{10}{9\omega_{0}^{2}}\alpha_{2}^{2}$$

Based on Equation (4), we found that α = −4749, which proves the dominant inherent quadratic nonlinearity of the arch, and thus it exhibits softening behaviour regardless of the DC voltage. We exploit this inherent nonlinearity of the arch for memory operation by modulating its nonlinearity by the DC voltage.

## 4. Results and Discussion

**Figure 3:**
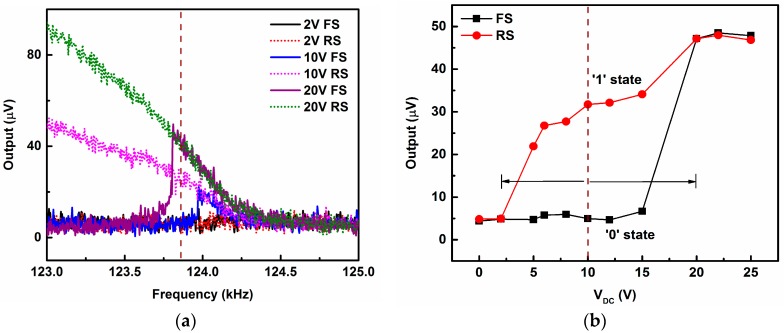
(**a**) Experimentally obtained frequency–response curves at different applied DC bias voltages while alternating current (AC) voltage is fixed at −5 dBm (0.125 V_rms_). Forward and backward sweeps (FS/BS) were performed to observe the frequency hysteresis cycle. Forward sweeps for all DC bias voltages are shown in solid lines, whereas the backward sweep curves are shown in dotted lines; (**b**) Response of the resonator with respect to the DC bias voltage at a fixed frequency (123.863 kHz) and fixed AC voltage (−5 dBm). The response shows a hysteretic behavior when the DC bias voltage is swept in forward (black line with square symbols) and backward directions (red line with circle symbols). The chosen operating point at 10 V, which has two states (“1” or “0”), and can be set or reset to memory states by momentarily increasing or decreasing the DC bias voltage, respectively.

Figure 3a shows the nonlinear response of the arch beam with different DC bias voltages. All curves are measured in forward and backward sweeps with a fixed AC voltage of −5 dBm (0.125 V_rms_). With an increase in the DC bias voltage, the edge of the resonance peak shifts towards lower resonance frequency due to the softening behavior. The responses demonstrate a noticeable hysteresis, creating a range of frequencies in which the beam is bi-stable and can be used to implement the proposed mechanical memory. We chose a frequency of 123.863 kHz (shown as a dashed line in Figure 3a) as the AC driving frequency for the demonstration of the memory operation.

Next we demonstrate the mechanical memory operation of the clamped–clamped shallow arch beam. It can be performed by modulating the drive frequency, the drive strength, or a combination of these across the hysteretic regime. These schemes were also utilized to implement nano-mechanical memory in clamped–clamped straight beams and cantilever micro-beams [5,6,7,8,9]. Here, the modulation of nonlinearity is achieved by varying the DC bias voltage. The principle is indicated by the arrows in Figure 3b, which shows the experimentally determined hysteretic behavior as a function of the DC bias voltage when the excitation frequency and AC excitation amplitude are fixed at 123.863 kHz and −5 dBm, respectively. From Figure 3b, it is clear that when the resonator is biased at 10 V, it has two states of vibration amplitude, depending on the direction of the sweep. Hereafter, we define the large and small amplitude vibrations as “1” and “0” memory states, respectively. The hysteretic region exists between 2 and 20 V DC voltages. Any voltage within this hysteretic region can be selected for operation of the memory device. Here we choose a voltage of 10 V as our operating point for implementation of the memory element.

### 4.1. Switching from Low Vibration Amplitude (“0”) to High Vibration Amplitude (“1”)

Initially, the low vibration amplitude (“0”) memory state is written by setting the DC bias voltage at 10 V, which corresponds to 4.94 µV of output response measured simultaneously by the network analyzer. To set the memory state to high vibration amplitude (“1”), the DC bias voltage is momentarily increased to 20 V. Any vibration amplitude above 21 µV is considered to be high vibration amplitude state (“1”). As can be seen in Figure 3b, at this DC bias voltage, the resonator has high vibration amplitude, which corresponds to 47.13 µV of output response and will maintain this state even when the DC voltage is reverted back to its operating voltage of 10 V. The resonator will be continued in its high vibration amplitude state indefinitely, until some changes are introduced in the operating conditions.

### 4.2. Switching from High Vibration Amplitude (“1”) to Low Vibration Amplitude (“0”)

Next, to access the “0” memory state again, the DC bias voltage is reduced to 2 V. In this case, any state below 6 µV is considered to be low vibration amplitude state (“0”). This will reset the arch beam to the low vibration amplitude state, which corresponds to 4.83 µV of output response at this operating condition, and it will retain this state when the DC bias voltage is increased to its operating point at 10 V.

### 4.3. Sequential Switching Operation between the Memory States

**Figure 4:**
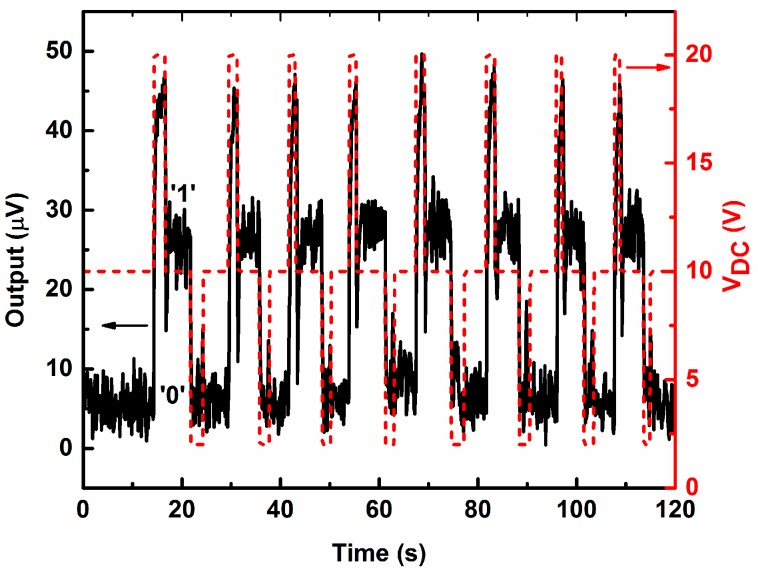
The sequential operation of the memory device. The black line represents the memory states (“1” or “0”). The **red** dashed line represents the waveform of the DC bias voltage used as write signal.

We also performed the sequential operation of the memory device as depicted in Figure 4. It shows the repeatability of the switching action between the two states. The black curve shows the memory states (“1” or “0”), whereas the red curve shows the writing operations. From the initial state of “0”, the resonator is set to a memory state “1” by a write signal (momentarily increasing the DC bias from 10 to 20 V). Again, by a subsequent write signal (momentarily decreasing DC bias voltage from 10 to 2 V), the memory state is reset to “0”. We observed controlled switching between the memory states during the write operations by the DC bias pulses.

### 4.4. Maximum Operating Speed and Energy Cost Per Switching between the Memory States

The maximum theoretical operating speed of this memory device can be estimated to be $f_{\mathrm{res}}/Q\approx 16\;\mathrm{Hz}$, which is same for the transition from 0 to 1 and 1 to 0. Another important aspect to consider is the energy cost of the memory operation. This can be estimated as the change in energy stored in the system due to the applied voltage necessary to switch the state of arch beam, *E ≈ CV*_DC_*V*_S_ ~ 10^−13^ J, where C is the overlap capacitance between the microbeam and the sense electrode, on the order of 10^−15^ F; *V*_DC_ = 10 V is the initial DC bias voltage; and *V*s = 10 V is the increase in the DC bias to switch the state of the memory device from memory state “0” to memory state “1”. The operating speed and energy cost of this proposed memory device can be further improved by shrinking its size and/or reducing the loaded quality factor. Shrinking the dimensions helps to increase the operation frequency and also significantly improves the areal density, which depends on second power of the length of the resonator, 1/*L*^2^. Additionally, the required voltage load to perform a memory operation could be reduced significantly by reducing the resonators’ dimensions and/or by working at high vacuum conditions.

## 5. Conclusions

We have demonstrated a dynamic memory device based on an intentionally fabricated arch shaped in-plane MEMS clamped–clamped resonator, actuated and sensed using standard electrostatic techniques. Softening behavior of the arch beam originating from the quadratic nonlinearity of its curvature is exploited for the first time for memory application. It is demonstrated that the two co-existing vibrational states on the bifurcation branch of a nonlinearly resonating arch beam can be effectively used as an information storage element, where the memory set/reset operations are performed by modulating the DC bias voltage. This silicon-based memory device is fabricated using a SOI fabrication process, works at room temperature and under modest vacuum conditions, and has the prospect for on-chip integrated system development to provide unprecedented advantages of miniaturization and integration.
